# Clinical Implication of Metformin in Relation to Diabetes Mellitus and Ovarian Cancer

**DOI:** 10.3390/biomedicines9081020

**Published:** 2021-08-16

**Authors:** Santosh Kumar Singh, Tejumola Apata, Shriti Singh, Melayshia McFadden, Rajesh Singh

**Affiliations:** 1 Department of Microbiology, Biochemistry, and Immunology, Morehouse School of Medicine, Atlanta, GA 30310, USA; sksingh@msm.edu (S.K.S.); apatatejumola@gmail.com (T.A.); MMcFadden@msm.edu (M.M.); 2 Department of Kriya Sharir, Institute of Medical Sciences, Banaras Hindu University, Varanasi 221005, India; singhshriti@gmail.com; 3 Cancer Health Equity Institute, Morehouse School of Medicine, Atlanta, GA 30310, USA

**Keywords:** metformin, diabetes mellitus, ovarian cancer

## Abstract

Since multiple reports established an association between diabetes mellitus and various cancers, emerging studies have surfaced to understand the effects of metformin as an anti-cancer agent. Although there was previous, but conflicting evidence, of a relationship between diabetes and ovarian cancer (OvCa), recent studies have supported this association. The mechanism of cancer development in patients with diabetes is likely to involve hyperglycemia, hyperinsulinemia, chronic inflammation, reactive oxygen species, regulation of cellular homeostasis, and activation of various pathways that lead to tumor cell proliferation. Preclinical evidence indicating that metformin, a medication commonly used to treat type 2 diabetes mellitus, may protect against OvCa. Metformin exerts anti-cancer properties by activating the MAPK pathway, inhibiting the PI3K/AKT/mTOR pathway, increasing tumor suppressor genes, inducing G2/M cycle arrest, and various other processes. Several studies have shown the efficacy of metformin as an adjunct with standard chemotherapeutic agents due to its synergistic effects on OvCa cells. This review highlights the epidemiologic evidence supporting a link between diabetes and OvCa, the fundamental molecular mechanism underlying carcinogenesis in patients with diabetes, the anti-cancer effects of metformin, and the need for further clinical investigations on combination therapies with metformin and standard chemotherapeutic agents for OvCa.

## 1. Introduction

In the United States, ovarian cancer (OvCa) is currently ranked as the fifth leading cause of death among malignancies, with estimated new cases of 21,410 and estimated deaths of 13,770 in 2021 [1]. The most common subtype of OvCa is derived from epithelial cells, and these mainly occur in post-menopausal women. In contrast, other types of OvCas, such as germ cells, occur more commonly in premenopausal women; stromal cell tumors can occur at any age [2]. Among all, high-grade serous ovarian cancer (HGSOC) is dominant and originates from surface epithelium, accounting for 70–80% of OvCa deaths [3]. Symptoms include abdominal swelling, bloating, pelvic pain, indigestion, altered bowel habits, and getting full easily [4]. Due to the ambiguity of its symptoms, most OvCas are diagnosed at advanced stages.

Several studies provide links between insulin resistance, hyperglycemia, diabetes mellitus (DM), and cancer [5,6]; however, the mechanism by which insulin resistance results in cancer is not fully understood. DM is a metabolic disease that results in hyperglycemia via insulin resistance or lack of insulin production. Various theories of the mechanism of carcinogenesis exist, and are further explored in this article.

Since OvCa and DM share common risk factors, including age, race, obesity, and diet, establishing a direct relationship between DM and OvCa can be difficult. However, several studies have suggested that DM increases the risk of cancer [5]. A systematic review and meta-analysis of 15 cohorts established a positive correlation between OvCa and DM [7].

Since an association between DM and OvCa has been established, some studies have explored how metformin, the most common drug used for treating type 2 diabetes (T2DM), is involved in pathways that promote carcinogenicity and induce apoptosis [8,9,10]. A retrospective study of patients with OvCa and concurrent T2DM treated with metformin showed that T2DM patients with OvCa receiving metformin had a longer progression-free survival and overall survival than T2DM patients with OvCa who did not take metformin [11]. When added to standard first-line chemotherapy, metformin also shows a change in outcome for non-diabetic patients with non-small cell lung cancer [12]. The present review summarizes evidence showing an association between DM and OvCa, molecular pathways that trigger ovarian carcinogenesis in DM, the effect of metformin as a chemotherapeutic agent, and the synergistic effects of metformin and standard chemotherapeutic regimens in the treatment of OvCa. We have gathered evidence to support the anti-cancer effect of metformin for OvCa. We trust that more research will be accomplished and that clinical trials will be performed to evaluate its efficacy in conjunction with other chemotherapeutic agents.

## 2. DM and Cancer Development

DM is associated with an increased risk of several types of malignancy, including breast, cervical, pancreatic, and colon cancer [13]. A retrospective case-control study of patients with cancer diagnosed after the development of T2DM relative to a control group showed that, among women, the most prevalent cancer sites were breast (36.5%), uterus (13.9%), colon with the rectum (9.6%), lung (5.2%), and stomach (4.4%). For men, the cancer sites were colon with the rectum (25.0%), prostate (13.6%), kidney (10.2%), lung (10.2%), and pancreas (9.1%) [13]. Although the mechanism of the link between DM is unclear, it has been proposed that hyperinsulinemia, hyperglycemia, signaling pathways via IGF-1 receptors, and other inflammatory markers are involved in the development of cancers. A prospective cohort study shows that cancer mortality is higher for patients with hyperinsulinemia than for those without it, even among non-obese patients [14]. Further, high fasting insulin levels are associated with poorer outcomes for women with early breast cancer [15].

### 2.1. Hyperinsulinemia Due to Insulin Resistance as Seen in T2DM

Hyperinsulinemia indirectly influences cancer development through the insulin-like growth factor (IGF) axis, which has various downstream mechanisms involving protein expression, glucose mechanism, and anti-apoptosis. Although most circulating IGF is produced via direct stimulation of hepatocytes by growth hormone, insulin is involved in pathways that downregulate the production of insulin-like growth factor-binding protein (IGFBP), thus increasing the circulating levels of IGF [16]. IGFBP serves as a regulatory protein by binding to IGF in circulation to prevent degradation of the ligand. The two major subsets of the IGF ligand, IGF-1 and IGF-2, bind to their receptors, IGF-1R and IGF-2R, to activate various pathways [17]. Once the ligand binds to the receptor, it is activated by autophosphorylation, leading to the activation of two separate pathways. The Ras/Raf/MEK/ERK pathway promotes cell proliferation, and the PI3K/AKT/mTOR pathway leads to protein synthesis, glucose metabolism, and decreased apoptosis. Insulin receptor substrate (IRS-1), which IGF-1R also activates via phosphorylation, binds to phosphoinositide 3-kinase (PI3K), leading to higher levels of phosphoinositol 3,4,5- triphosphate (PIP3) [17].

PIP3 activates AKT, which leads to activation of the anti-apoptotic markers BCL-2 and Bcl-xl and inhibition of pro-apoptotic proteins such as BAD [17,18]. FOXO gene expression is also inhibited by activation of AKT and the tumor suppressor gene P27. In the other pathway, after phosphorylation of Shc by IGF-1R, a series of phosphorylation events occur to activate Raf, MEK, and ERK, resulting in the activation of ELK1, which regulates the expression of genes leading to cell proliferation [18].

Although insulin affects this pathway indirectly by increasing levels of IGF-1, insulin itself can activate IRS-1 directly, thus activating the pathway [19]. At physiological concentrations, insulin increases the rate of DNA, RNA, and protein synthesis in MCF-7 human breast cancer cells [20]. However, for diabetic patients, hyperinsulinemia alone cannot be the driving force towards oncogenesis, at least not for all tumor cells. A study of mammary tumors of rats, for which type 1 diabetes mellitus (T1DM) was induced by injecting rats with 7,12-dimethylbenz(a)anthracene, destroyed β-pancreatic cells, and subsequent hyperglycemia led to mammary tumor regression [21]. This study showed that lack of insulin in rats with induced DM led to tumor regression. This contradicts a study showing that induction of T1DM by injecting mice with the β-cell toxin streptozotocin led to larger tumors and metastasis than those in normoglycemic mice [22], suggesting that hyperglycemia in the absence of hyperinsulinemia, as seen in T2DM, is also involved in cancer development.

### 2.2. Hyperglycemia as Seen in T1DM and T2DM and Its Effect on Cancer Development

The role of hyperglycemia in developing hyper-proliferating cells that lead to oncogenesis is explained by the Warburg effect [23]. Differentiated cells metabolize glucose to high-energy molecules such as NADH and pyruvate via the glycolytic pathway, generating ATP production via the tricarboxylic acid cycle and oxidative phosphorylation in the presence of oxygen. Increased glucose consumption is used as a carbon source for anabolic processes needed to support cell proliferation. An increase in breast cancer cell proliferation and activation of oncogenic signaling pathways are associated with media glucose concentrations greater than 5 mM [24]. In addition, elevated glucose levels meet the energy demand for OvCa growth; therefore, a low circulating glucose concentration may be a constraining factor in cell metabolism [22].

Chronic hyperglycemia leads to oxidative stress by producing reactive oxygen species (ROS), which cause DNA mutations. Therefore, ROS is essential in the initiation and progression of carcinogenesis [25]. ROS activates a signaling cascade that leads to the degradation of I-KB (an inhibitor of NF-κB). After activation, NF-κB travels to the cell’s nucleus to induce transcription of pro-inflammatory genes [26]. It also leads to activation of the MAPK pathway, which induces transcription of genes that promote cell proliferation, survival, and metastasis [27].

Protein glycation and formation of advanced glycation end-products (AGEs) are involved in the pathogenesis of diabetic complications, including retinopathy, nephropathy, neuropathy, cardiomyopathy, along with diseases, including rheumatoid arthritis, osteoporosis, and aging [28]. AGEs, developed as a consequence of long-standing hyperglycemia, are formed by complex, non-enzymatic chemical reactions between sugars and amino-containing molecules such as proteins, amino acids, amino lipids, and nucleic acids. They stimulate the generation of inflammatory cytokines (TNF-α, interleukin-1, interleukin-6), growth factors (TGF-b1, IGF-1), metalloproteases, chemokines, and other bioactive molecules [26]. The effect of AGEs on cell proliferation, migration, and invasion has been demonstrated for the breast cancer cell line MDA-MB-231; AGEs enhance MMP-9 activity via activation of the transmembrane protein RAGE (receptor of advanced glycated end-products) [29].

### 2.3. Chronic Inflammation as Seen in DM and Its Relationship with the Development of OvCa

There is an association between T2DM and cancer development related to a pathogenic state of chronic inflammation. The state of poor metabolic control causes a permanent pro-inflammatory condition that promotes inflammatory cytokines such as IL-6, TNFα, LPS, plasminogen activator inhibitor-1, and other proteins involved in cancer development and progression [30,31]. In diabetic patients, binding of these pro-inflammatory ligands to their receptors leads to activation of signaling pathways that dissociate NF-κB from its inhibitor IκB proteins [31]. NF-κB signaling is involved in pathways leading to cell differentiation, inflammation, proliferation, and apoptosis [32].

IL-6, a cytokine in OvCa cells, initiates signaling pathways, leading to tumor proliferation, angiogenesis, and chemo-resistance [33]. Activated pathways include Janus kinase/STAT [34,35,36], mitogen-activated protein kinase [37], and PI3K/AKT [37]. JAK2 is activated in low- and high-grade ovarian carcinomas, and it is expressed in normal ovaries and in benign, borderline, and histological-grade tumors [38], which suggests an essential role for this factor in OvCa carcinogenesis.

## 3. Theories Regarding the Biological Link between DM and OvCa

Most previous epidemiological studies [39,40,41] report no increased risk of OvCa among patients with DM; but some show a borderline significance of associated risk [42]. For this reason, the relationship between DM and OvCa was unclear until recent systematic reviews and meta-analyses of observational and cohort studies showed a clear association between DM and OvCa [7,43,44]. Although molecular mechanisms for the link between DM and OvCa are yet to be uncovered, there are theories to explain this relationship, including the IGF-I/PI3K/AKT/mTOR pathway used to explain pathogenic connections between DM and other cancers. The KCl co-transport (KCC) protein is essential for developing gynecological cancers; it is involved in the downstream pathways towards cell proliferation and depends on activation of the IGF-1 pathway [45]. The KCC protein, which exists in three isoforms KCC1, KCC3, and KCC4, regulates cell volume and the influx and efflux ions necessary to maintain the structural integrity [45]. Because cancer cells usually have a higher growth rate and migration than normal cells, they possess mechanisms to accommodate their volume expansion. Therefore, overexpression of these KCC transporters on cancer cells maintains homeostasis of the cell’s volume. KCC activity is enhanced after activating the IGF-receptor via downstream activation of MAPK/Erk1/2 and PI3K signaling pathways [46]. Once these pathways are activated, mRNA levels of KCC1, KCC3, and KCC4 increase. When an inhibitor blocks KCC activity, cellular invasion and proliferation are reduced.

In addition to regulating cell volume, KCC activity is also associated with cancer invasion and metastasis by activating matrix metalloproteinases (MMPs) [47], enzymes involved in collagen degradation in basement membranes [48]. The KCC4 isoform, isolated from metastatic OvCa cells, interacts with ezrin, a protein involved in epithelial cell migration and adhesion [49].

## 4. Antineoplastic Mechanisms of Metformin on OvCa Cells

Recent evidence supports an antineoplastic effect of metformin on cancer cells. There have been several reports on the effectiveness of metformin on OvCa. Although several studies have investigated mechanistic pathways of metformin as an anti-cancer agent, few have revealed targets of the drug in OvCa cells. The molecular mechanisms for the association between DM and the development of OvCa are still being uncovered.

Metformin, a biguanide derivative, is considered a first-line anti-diabetic drug for managing T2DM. It is reported to be associated with a reduced risk of cancer for patients with T2DM [50]. Although various mechanisms have been considered in understanding its impact on DM and cancer, the most common theory regarding the action of metformin in reducing hyperglycemia and its anti-cancer effects is via activation of the kinase B1 (LKB1)-AMPK pathway in the liver [51].

AMPK, a heterotrimeric protein kinase that regulates energy metabolism, is activated by oxidative stress, ischemia, hypoxia, and low glucose. Metformin leads to AMPK activation by inhibiting complex 1 of the respiratory chain in the mitochondria, leading to a high AMP/ATP ratio due to a low ATP concentration and elevated conversion of ADP to AMP. Once activated, AMPK leads to various downstream effects, such as activation of tumor suppressor genes P52 and FOXO3a and to inhibition of mTOR, which regulates cell growth and proliferation [52]. Activation of AMPK also inhibits the PI3K/AKT pathway.

The PI3K/AKT/mTOR pathway, once activated by IGF-I and other growth factors, leads to a series of signaling cascades that promote cell proliferation, growth, invasion, and metastases of various cancers, including OvCa [53]. This pathway is activated in approximately 70% of OvCa cases [54]. Metformin inhibits this pathway via interaction with cysteine-rich 61 (Cyr61), a member of the CCN family of growth factors (Cyr61/CTGF/Nov) that is overexpressed in cancers, including OvCas [55]. In OVCAR-3 OvCa cells, overexpression of Cyr61 enhances cell proliferation by inhibiting carboplatin-induced apoptosis, decreasing expression of the pro-apoptotic marker Bax, and increasing expression of anti-apoptotic markers Bcl-xl, Mcl-1, and Blc-2 [56]. It also downregulates the tumor suppressor P53 and upregulates NF-KB. Metformin downregulates the protein expression of Cyr61, leading to downregulation of pAKT and mTOR, a process that favors apoptosis [55].

In a concentration-dependent manner, metformin induces G2/M cell cycle arrest, thus increasing the numbers of cancer cells in the G2 phase [57]. In addition, it causes DNA damage in OvCa cells, likely due to increased accumulation of intracellular ROS [57].

**Figure 1:**
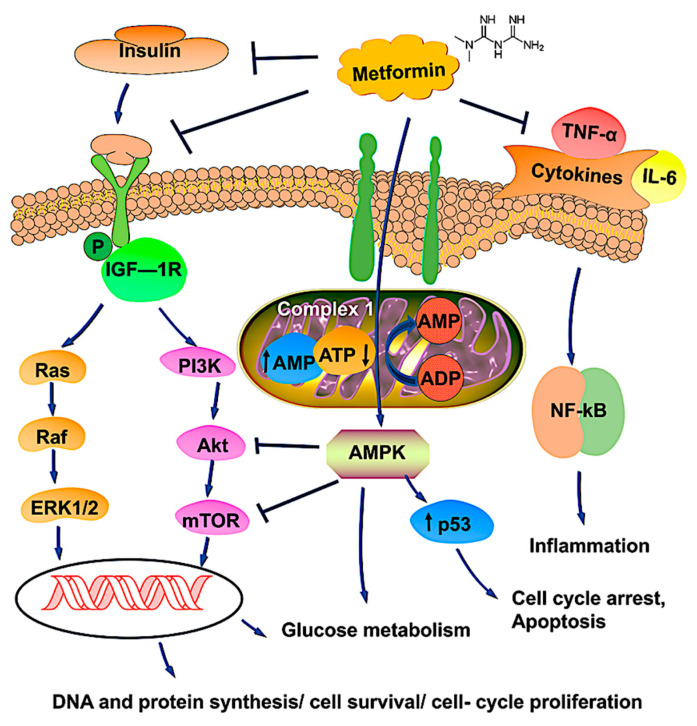
Schematic representation of metformin’s possible mechanism of action in OvCa therapy. Metformin, a first-line anti-diabetic drug, is likely to be associated with a reduced risk of cancer for those with T2DM. The most common pathway involves the activation of AMPK, which regulates energy metabolism by modulating complex 1 of the respiratory chain in mitochondria by changes in the AMP/ATP ratio. Activated AMPK upregulates the tumor suppressor gene p53, which induces apoptosis and cell cycle arrest. Metformin binds with insulin, IGF, and cytokine receptors and modulates pathways involved in tumor progression. Upon binding, metformin inhibits the PI3K/AKT/mTOR and Ras/Raf/ERK pathways, leading to reductions of DNA synthesis and cell proliferation, thereby causing tumor cell death. Metformin, through AMPK activation and mTOR inhibition, could increase glucose uptake and glycolysis. Overall, metformin promotes phosphorylation, blocking nuclear translocation and impairing gene transcription, processes involved in cell survival, gluconeogenesis, and protein synthesis. The arrows ↑ and ↓ indicates upregulation and downregulation, respectively.

Chronic inflammation contributes to pathways that enhance cell proliferation and tumorigenesis. A recent study shows that the OvCa tumor stroma of metformin-treated patients exhibits lower IL6 expression via inhibition of NF-KB [58]. This is a relevant association because IL6 is an inflammatory marker involved in pathogenic pathways in the carcinogenesis of OvCa and inflammation-mediated chemo-resistance. A schematic representation of metformin and its possible mechanisms is shown in Figure 1.

## 5. The Future of Metformin as an Adjunct for OvCa Therapy

Metformin has been evaluated for its potential synergistic effects with traditional chemotherapeutic agents for the treatment of OvCa. The standard chemotherapy regime involves a combination of a platinum-based agent such as cisplatin or carboplatin and a taxane such as docetaxel or paclitaxel. Metformin enhances the inhibitory effects of cisplatin on epithelial OvCa in vitro and in vivo [59]. The mechanism proposed here is that metformin inhibits the TGFB-1 pathway, thus decreasing the TGFB-1-induced epithelial-mesenchymal transition, N-cadherin, and MMP, all involved in cancer metastasis. Metformin also has synergistic effects with paclitaxel, as it increases the activity of this chemotherapeutic agent. The combination of metformin and paclitaxel produced a 60% reduction in tumor weight compared to 40% and 42% reductions seen for paclitaxel alone and metformin alone, respectively [60]. Other studies with OvCa cells have also shown synergistic effects of metformin with chemotherapeutic agents such as dichloroacetate [60] and trametinib [61].

A next step to introducing metformin as a nonadjunct therapy to standard chemotherapy agents would be its success in a prospective clinical trial. To the best of our knowledge, only Shahid Faghihi Hospital in Iran has completed a randomized control trial to demonstrate the efficacy of metformin in conjunction with the standard carboplatin-paclitaxel regime after cytoreduction surgery for epithelial OvCa (clinical trials code IRCT2016022726788N1). Patients who underwent total abdominal hysterectomy and bilateral salpingo-oophorectomy diagnosed with epithelial OvCa were considered for this study. A total of 41 patients from each control and intervention group was used to participate. The study results showed a 13.3% 4-year recurrence rate for the study group versus a 67.5% recurrence rate for the control group. The mean survival of patients in the study group was also higher than in the control group [62]. Although this randomized control study supports the hypothesis that metformin increases the cytotoxic effects of standard chemotherapy agents on OvCa, a limitation is its small sample size (41 patients from each group). According to the NIH https://clinicaltrials.gov (accessed on 23 July 2021), there are six ongoing clinical trials (Table 1) to evaluate the effects of metformin in patients receiving chemotherapy for OvCa. These studies, however, are either still recruiting or are in phase 1 or 2; therefore, no results or conclusions have been reported. However, side effects such as gastrointestinal disorders [63], diarrhea, dyspepsia, and flatulence [64] are the major limitations reported in the clinical trials.

Further, metformin has been shown a potential role in immunotherapy, particularly in checkpoint mechanisms, represented by the programmed cell death protein 1 (PD-1)/programmed cell death-ligand 1 (PD-L1) interaction. A study confirms that a patient who received metformin plus anti-PD1 alters immunotherapy resistance by preventing PD-1+/CD8+ T-cell infiltrates [65]. In a separate survey, metformin enhances the efficacy of PD-1 blockade by reducing the tumor hypoxia in a mouse model [66]. Since T cells activity in the tumor microenvironment has remained the main goal for US Food and Drug Administration (FDA) approval on the drugs; Liu et al. [67] displayed several approaches where metformin can enhance T-cell immunity by (1) countering the suppressed state of CD8TILs via AMPK-mTOR signaling, (2) alleviating the intra-tumoral hypoxic state of the tumor microenvironment, and (3) regulating the state of the tumor immune microenvironment (TIME) by downregulating the expression of macrophages, CD39 + CD73 + MSDC, tumor-infiltrating CD4 + CD25 + Treg cells, and upregulation of CD+ T cells, and metabolic reprogramming of Treg cells. A recent report established metformin can holds anti-tumor activity in endometrial cancer by inhibiting the expression of PD-L1 in an AMPK-dependent manner [68]. Some clinical trial observations indicate metformin with checkpoint inhibitors (nivolumab, pembrolizumab, and durvalumab) is effective in treating tumors [67]. Indeed, we may overlook various facets where metformin modulates complex immune mechanisms. Therefore, more research is needed to understand the tool before recommending routine therapy in cancer, including OvCa.

## 6. Conclusions

The findings reported in this review provide evidence showing the various effects of metformin as an anti-cancer agent, especially as an adjunct with existing standard chemotherapeutic drugs for OvCa. Since there has been only one clinical trial with results to support the use of metformin as a nonadjunct agent in combination with other drugs, we recommend that more research studies be performed to support these findings.

## Figures and Tables

**Table 1:** Updates on clinical trials of metformin and OvCa.

Disease Condition	Treatment	Summary	Trial Phase Status	Clinical Trial Number
Advanced stage OvCa	Metformin with carboplatin/paclitaxel	mTOR pathway inhibition, p53-induced apoptosis.	Phase1	NCT02312661
Advanced stage OvCa	Paclitaxel, carboplatin, and oral metformin	Increased synergy without compromising patient tolerability.	Phase 2	NCT02437812
Advanced epithelial OvCa in Stages IIIa–-IV	Metformin, acetylsalicylic acid, olaparib, and letrozole	Women with advanced (stage IIIa-IV) OvCa of the histologic subtype high-grade serous carcinoma (HGSOC) are going through a diagnostic laparoscopy. They will receive treatment with a study agent for 10–14 days before surgery. The study is randomized and unblinded.	Early Phase 1	NCT03378297
Complex endometrial hyperplasia with atypia grade 1 endometrial endometrioid adenocarcinoma	Levonorgestrel and metformin	Metformin is an effective treatment for early-stage endometrial cancer and endometrial hyperplasia with atypia.	Phase 2	NCT01686126
Ovarian, fallopian tube, and primary peritoneal cancer	Metformin	To determine if metformin administered in combination with chemotherapy to women with advanced ovarian, primary peritoneal, or fallopian tube cancer will improve recurrence-free survival at 18 months compared to controls.	Phase 2	NCT01579812
Cancer	Metformin, atorvastatin, doxycycline, and mebendazole	To determine the effectiveness of a regimen of selected metabolic treatments for patients with cancer in a real-world setting and to conduct exploratory analysis on the relationship between the degree of response and changes in biochemical markers (such as glucose and lipid levels).	Phase 3	NCT02201381

## References

[1] Siegel R.L., Miller K.D., Fuchs H.E., Jemal A. (2021). Cancer Statistics, 2021. CA Cancer J. Clin..

[2] Doubeni C.A., Doubeni A.R., Myers A.E. (2016). Diagnosis and Management of Ovarian Cancer. Am. Fam. Physician.

[3] Lisio M.A., Fu L., Goyeneche A., Gao Z.H., Telleria C. (2019). High-Grade Serous Ovarian Cancer: Basic Sciences, Clinical and Therapeutic Standpoints. Int. J. Mol. Sci..

[4] Gajjar K., Ogden G., Mujahid M.I., Razvi K. (2012). Symptoms and risk factors of ovarian cancer: A survey in primary care. ISRN Obstet. Gynecol..

[5] Shlomai G., Neel B., LeRoith D., Gallagher E.J. (2016). Type 2 Diabetes Mellitus and Cancer: The Role of Pharmacotherapy. J. Clin. Oncol..

[6] Li A., Qiu M., Zhou H., Wang T., Guo W. (2017). PTEN, Insulin Resistance and Cancer. Curr. Pharm. Des..

[7] Zhang D., Li N., Xi Y., Zhao Y., Wang T. (2017). Diabetes mellitus and risk of ovarian cancer. A systematic review and meta-analysis of 15 cohort studies. Diabetes Res. Clin. Pract..

[8] Romero I.L., McCormick A., McEwen K.A., Park S., Karrison T., Yamada S.D., Pannain S., Lengyel E. (2012). Relationship of type II diabetes and metformin use to ovarian cancer progression, survival, and chemosensitivity. Obstet. Gynecol..

[9] Rattan R., Giri S., Hartmann L.C., Shridhar V. (2011). Metformin attenuates ovarian cancer cell growth in an AMP-kinase dispensable manner. J. Cell Mol. Med..

[10] Zhu J., Zheng Y., Zhang H., Sun H. (2016). Targeting cancer cell metabolism: The combination of metformin and 2-Deoxyglucose regulates apoptosis in ovarian cancer cells via p38 MAPK/JNK signaling pathway. Am. J. Transl. Res..

[11] Wang S.B., Lei K.J., Liu J.P., Jia Y.M. (2017). Continuous use of metformin can improve survival in type 2 diabetic patients with ovarian cancer: A retrospective study. Medicine.

[12] Marrone K.A., Zhou X., Forde P.M., Purtell M., Brahmer J.R., Hann C.L., Kelly R.J., Coleman B., Gabrielson E., Rosner G.L. (2018). A Randomized Phase II Study of Metformin plus Paclitaxel/Carboplatin/Bevacizumab in Patients with Chemotherapy-Naïve Advanced or Metastatic Nonsquamous Non-Small Cell Lung Cancer. Oncologist.

[13] Dąbrowski M., Szymańska-Garbacz E., Miszczyszyn Z., Dereziński T., Czupryniak L. (2016). Risk factors for cancer development in type 2 diabetes: A retrospective case-control study. BMC Cancer.

[14] Tsujimoto T., Kajio H., Sugiyama T. (2017). Association between hyperinsulinemia and increased risk of cancer death in nonobese and obese people: A population-based observational study. Int. J. Cancer.

[15] Goodwin P.J., Ennis M., Pritchard K.I., Trudeau M.E., Koo J., Madarnas Y., Hartwick W., Hoffman B., Hood N. (2002). Fasting insulin and outcome in early-stage breast cancer: Results of a prospective cohort study. J. Clin. Oncol..

[16] Arcidiacono B., Iiritano S., Nocera A., Possidente K., Nevolo M.T., Ventura V., Foti D., Chiefari E., Brunetti A. (2012). Insulin resistance and cancer risk: An overview of the pathogenetic mechanisms. Exp. Diabetes Res..

[17] Denduluri S.K., Idowu O., Wang Z., Liao Z., Yan Z., Mohammed M.K., Ye J., Wei Q., Wang J., Zhao L. (2015). Insulin-like growth factor (IGF) signaling in tumorigenesis and the development of cancer drug resistance. Genes Dis..

[18] Zha J., Lackner M.R. (2010). Targeting the insulin-like growth factor receptor-1R pathway for cancer therapy. Clin. Cancer Res..

[19] Pollak M. (2008). Insulin and insulin-like growth factor signalling in neoplasia. Nat. Rev. Cancer.

[20] Osborne C.K., Bolan G., Monaco M.E., Lippman M.E. (1976). Hormone responsive human breast cancer in long-term tissue culture: Effect of insulin. Proc. Natl. Acad. Sci. USA.

[21] Heuson J.C., Legros N. (1972). Influence of insulin deprivation on growth of the 7,12-dimethylbenz(a)anthracene-induced mammary carcinoma in rats subjected to alloxan diabetes and food restriction. Cancer Res..

[22] Kellenberger L.D., Petrik J. (2018). Hyperglycemia promotes insulin-independent ovarian tumor growth. Gynecol. Oncol..

[23] Vander Heiden M.G., Cantley L.C., Thompson C.B. (2009). Understanding the Warburg effect: The metabolic requirements of cell proliferation. Science.

[24] Wahdan-Alaswad R., Fan Z., Edgerton S.M., Liu B., Deng X.S., Arnadottir S.S., Richer J.K., Anderson S.M., Thor A.D. (2013). Glucose promotes breast cancer aggression and reduces metformin efficacy. Cell Cycle.

[25] Duan W., Shen X., Lei J., Xu Q., Yu Y., Li R., Wu E., Ma Q. (2014). Hyperglycemia, a neglected factor during cancer progression. Biomed Res. Int..

[26] Negre-Salvayre A., Salvayre R., Augé N., Pamplona R., Portero-Otín M. (2009). Hyperglycemia and glycation in diabetic complications. Antioxid. Redox Signal..

[27] Weinberg F., Hamanaka R., Wheaton W.W., Weinberg S., Joseph J., Lopez M., Kalyanaraman B., Mutlu G.M., Budinger G.R., Chandel N.S. (2010). Mitochondrial metabolism and ROS generation are essential for Kras-mediated tumorigenicity. Proc. Natl. Acad. Sci. USA.

[28] Singh V.P., Bali A., Singh N., Jaggi A.S. (2014). Advanced glycation end products and diabetic complications. Korean J. Physiol. Pharmacol..

[29] Sharaf H., Matou-Nasri S., Wang Q., Rabhan Z., Al-Eidi H., Al Abdulrahman A., Ahmed N. (2015). Advanced glycation endproducts increase proliferation, migration and invasion of the breast cancer cell line MDA-MB-231. Biochim. Biophys. Acta.

[30] Shikata K., Ninomiya T., Kiyohara Y. (2013). Diabetes mellitus and cancer risk: Review of the epidemiological evidence. Cancer Sci..

[31] Andreasen A.S., Kelly M., Berg R.M., Møller K., Pedersen B.K. (2011). Type 2 diabetes is associated with altered NF-κB DNA binding activity, JNK phosphorylation, and AMPK phosphorylation in skeletal muscle after LPS. PLoS ONE.

[32] Xia L., Tan S., Zhou Y., Lin J., Wang H., Oyang L., Tian Y., Liu L., Su M., Wang H. (2018). Role of the NFκB-signaling pathway in cancer. Onco. Targets Ther..

[33] Browning L., Patel M.R., Horvath E.B., Tawara K., Jorcyk C.L. (2018). IL-6 and ovarian cancer: Inflammatory cytokines in promotion of metastasis. Cancer Manag. Res..

[34] Heinrich P.C., Behrmann I., Müller-Newen G., Schaper F., Graeve L. (1998). Interleukin-6-type cytokine signalling through the gp130/Jak/STAT pathway. Biochem. J..

[35] Watanabe S., Mu W., Kahn A., Jing N., Li J.H., Lan H.Y., Nakagawa T., Ohashi R., Johnson R.J. (2004). Role of JAK/STAT pathway in IL-6-induced activation of vascular smooth muscle cells. Am. J. Nephrol..

[36] Beauchamp M.C., Yasmeen A., Knafo A., Gotlieb W.H. (2010). Targeting insulin and insulin-like growth factor pathways in epithelial ovarian cancer. J. Oncol..

[37] Kagan P., Sultan M., Tachlytski I., Safran M., Ben-Ari Z. (2017). Both MAPK and STAT3 signal transduction pathways are necessary for IL-6-dependent hepatic stellate cells activation. PLoS ONE.

[38] Colomiere M., Ward A.C., Riley C., Trenerry M.K., Cameron-Smith D., Findlay J., Ackland L., Ahmed N. (2009). Cross talk of signals between EGFR and IL-6R through JAK2/STAT3 mediate epithelial-mesenchymal transition in ovarian carcinomas. Br. J. Cancer.

[39] Adler A.I., Weiss N.S., Kamb M.L., Lyon J.L. (1996). Is diabetes mellitus a risk factor for ovarian cancer? A case-control study in Utah and Washington (United States). Cancer Causes Control.

[40] Gapstur S.M., Patel A.V., Diver W.R., Hildebrand J.S., Gaudet M.M., Jacobs E.J., Campbell P.T. (2012). Type II diabetes mellitus and the incidence of epithelial ovarian cancer in the cancer prevention study-II nutrition cohort. Cancer Epidemiol. Biomark. Prev..

[41] La Vecchia C., Negri E., Franceschi S., D’Avanzo B., Boyle P. (1994). A case-control study of diabetes mellitus and cancer risk. Br. J. Cancer.

[42] Inoue M., Iwasaki M., Otani T., Sasazuki S., Noda M., Tsugane S. (2006). Diabetes mellitus and the risk of cancer: Results from a large-scale population-based cohort study in Japan. Arch. Intern. Med..

[43] Lee J.Y., Jeon I., Kim J.W., Song Y.S., Yoon J.M., Park S.M. (2013). Diabetes mellitus and ovarian cancer risk: A systematic review and meta-analysis of observational studies. Int. J. Gynecol. Cancer.

[44] Wang L., Wang L., Zhang J., Wang B., Liu H. (2017). Association between diabetes mellitus and subsequent ovarian cancer in women: A systematic review and meta-analysis of cohort studies. Medicine.

[45] Lauf P.K., Adragna N.C. (2000). K-Cl cotransport: Properties and molecular mechanism. Cell Physiol. Biochem..

[46] Shen M.R., Lin A.C., Hsu Y.M., Chang T.J., Tang M.J., Alper S.L., Ellory J.C., Chou C.Y. (2004). Insulin-like growth factor 1 stimulates KCl cotransport, which is necessary for invasion and proliferation of cervical cancer and ovarian cancer cells. J. Biol. Chem..

[47] Shen M.R., Chou C.Y., Hsu K.F., Hsu Y.M., Chiu W.T., Tang M.J., Alper S.L., Ellory J.C. (2003). KCl cotransport is an important modulator of human cervical cancer growth and invasion. J. Biol. Chem..

[48] Jabłońska-Trypuć A., Matejczyk M., Rosochacki S. (2016). Matrix metalloproteinases (MMPs), the main extracellular matrix (ECM) enzymes in collagen degradation, as a target for anticancer drugs. J. Enzyme Inhib. Med. Chem..

[49] Chen Y.F., Chou C.Y., Wilkins R.J., Ellory J.C., Mount D.B., Shen M.R. (2009). Motor protein-dependent membrane trafficking of KCl cotransporter-4 is important for cancer cell invasion. Cancer Res..

[50] Evans J.M., Donnelly L.A., Emslie-Smith A.M., Alessi D.R., Morris A.D. (2005). Metformin and reduced risk of cancer in diabetic patients. BMJ.

[51] Rena G., Hardie D.G., Pearson E.R. (2017). The mechanisms of action of metformin. Diabetologia.

[52] Ikhlas S., Ahmad M. (2017). Metformin: Insights into its anticancer potential with special reference to AMPK dependent and independent pathways. Life Sci..

[53] Li H., Zeng J., Shen K. (2014). PI3K/AKT/mTOR signaling pathway as a therapeutic target for ovarian cancer. Arch. Gynecol. Obstet..

[54] Gasparri M.L., Bardhi E., Ruscito I., Papadia A., Farooqi A.A., Marchetti C., Bogani G., Ceccacci I., Mueller M.D., Benedetti Panici P. (2017). PI3K/AKT/mTOR Pathway in Ovarian Cancer Treatment: Are We on the Right Track?. Geburtshilfe Frauenheilkd.

[55] Zhang F., Chen H., Du J., Wang B., Yang L. (2018). Anticancer Activity of Metformin, an Antidiabetic Drug, Against Ovarian Cancer Cells Involves Inhibition of Cysteine-Rich 61 (Cyr61)/Akt/Mammalian Target of Rapamycin (mTOR) Signaling Pathway. Med. Sci. Monit..

[56] Lee K.B., Byun H.J., Park S.H., Park C.Y., Lee S.H., Rho S.B. (2012). CYR61 controls p53 and NF-κB expression through PI3K/Akt/mTOR pathways in carboplatin-induced ovarian cancer cells. Cancer Lett..

[57] Fu Y.L., Zhang Q.H., Wang X.W., He H. (2017). Antidiabetic drug metformin mitigates ovarian cancer SKOV3 cell growth by triggering G2/M cell cycle arrest and inhibition of m-TOR/PI3K/Akt signaling pathway. Eur. Rev. Med. Pharmacol. Sci..

[58] Xu S., Yang Z., Jin P., Yang X., Li X., Wei X., Wang Y., Long S., Zhang T., Chen G. (2018). Metformin Suppresses Tumor Progression by Inactivating Stromal Fibroblasts in Ovarian Cancer. Mol. Cancer Ther..

[59] Zheng Y., Zhu J., Zhang H., Liu Y., Sun H. (2018). Metformin inhibits ovarian cancer growth and migration in vitro and in vivo by enhancing cisplatin cytotoxicity. Am. J. Transl. Res..

[60] Lengyel E., Litchfield L.M., Mitra A.K., Nieman K.M., Mukherjee A., Zhang Y., Johnson A., Bradaric M., Lee W., Romero I.L. (2015). Metformin inhibits ovarian cancer growth and increases sensitivity to paclitaxel in mouse models. Am. J. Obstet. Gynecol..

[61] Mert I., Chhina J., Allo G., Dai J., Seward S., Carey M.S., Llaurado M., Giri S., Rattan R., Munkarah A.R. (2017). Synergistic effect of MEK inhibitor and metformin combination in low grade serous ovarian cancer. Gynecol. Oncol..

[62] Hamedi B., Khalili A., Roozmeh S., Namazi G., Saraf Z. (2018). Combination of Metformin and Chemotherapy Decreases the Recurrence Rates of Epithelial Ovarian Cancers: A Randomized Clinical Trial. Int. J. Cancer Manag..

[63] Facchinetti F., Orru B., Grandi G., Unfer V. (2019). Short-term effects of metformin and myo-inositol in women with polycystic ovarian syndrome (PCOS): A meta-analysis of randomized clinical trials. Gynecol. Endocrinol..

[64] Hameed M., Khan K., Salman S., Mehmood N. (2017). Dose Comparison and Side Effect Profile of Metformin Extended Release Versus Metformin Immediate Release. J. Ayub Med. Coll. Abbottabad.

[65] Haikala H.M., Anttila J.M., Marques E., Raatikainen T., Ilander M., Hakanen H., Ala-Hongisto H., Savelius M., Balboa D., Von Eyss B. (2019). Pharmacological reactivation of MYC-dependent apoptosis induces susceptibility to anti-PD-1 immunotherapy. Nat. Commun..

[66] Scharping N.E., Menk A.V., Whetstone R.D., Zeng X., Delgoffe G.M. (2017). Efficacy of PD-1 Blockade Is Potentiated by Metformin-Induced Reduction of Tumor Hypoxia. Cancer Immunol. Res..

[67] Liu W., Wang Y., Luo J., Liu M., Luo Z. (2020). Pleiotropic Effects of Metformin on the Antitumor Efficiency of Immune Checkpoint Inhibitors. Front. Immunol..

[68] Xue J., Li L., Li N., Li F., Qin X., Li T., Liu M. (2019). Metformin suppresses cancer cell growth in endometrial carcinoma by inhibiting PD-L1. Eur. J. Pharmacol..

